# Uptake of COVID-19 booster shot among healthcare workers: A mediation analysis approach

**DOI:** 10.3389/fpubh.2022.1033473

**Published:** 2022-10-05

**Authors:** Shazia Rehman, Erum Rehman, Zhang Jianglin

**Affiliations:** ^1^Department of Biomedical Sciences, Pak-Austria Fachhochschule, Institute of Applied Sciences and Technology, Haripur, Pakistan; ^2^Department of Mathematics, Nazarbayev University, Nur-Sultan, Kazakhstan; ^3^Department of Dermatology, Shenzhen People's Hospital, The Second Clinical Medical College, Jinan University, The First Affiliated Hospital, Southern University of Science and Technology, Shenzhen, China; ^4^Candidate Branch of National Clinical Research Center for Skin Diseases, Shenzhen, China

**Keywords:** vaccine hesitation, healthcare workers, decision regret, willingness, vaccination adverse reaction, booster shots, COVID-19

## Abstract

Since the thrust of previous research investigations has been on people's willingness to get immunized against the COVID-19 infection, the underpinning principle of compliance has received very little attention. Addressing the possible drivers and mechanisms influencing vaccine acceptance may provide significant insights for limiting the pandemic. In response, we intend to investigate the influence of decision regret and the consequences of post-vaccination adverse effects on the inclination to undertake booster shots. An electronic survey that was self-administered was conducted in Rawalpindi, Pakistan. The questionnaire was completed by 1,369 participants, with a response rate of 41%. 1,343 of them (98.10%) had received both doses of the COVID-19 vaccination. Besides, the present research has also adopted a mediation model. Our findings demonstrate that unfavorable vaccination responses in healthcare workers significantly affect their likelihood of receiving booster shots. Interestingly, healthcare workers who had adverse experiences after being immunized were more prone to regret their prior immunization decisions, which in response affected their decision to get a booster shot. The motivation to receive the booster dosage and adverse post-vaccination responses were mediated by decision regret. The outcomes suggested indissociable connections between unfavorable vaccination responses, decision regret, and the likelihood of receiving a booster shot. To strengthen immunization acceptance intent and enhance the likelihood of receiving COVID-19 booster shots, it is recommended that awareness of these post-vaccination adverse events be extensively integrated into immunization awareness programs and policy measures supporting booster doses.

## Introduction

According to the World Health Organization, Pakistan experienced 1,567,147 confirmed cases of COVID-19 from January 3, 2020, to August 26, 2022, with 30,559 fatalities (1). Pakistan is presently coping with the fifth cycle triggered by the new variant, Omicron. In November 2021, South Africa and Botswana reported the existence of the latest coronavirus variant. In subsequent days, it has taken over as Pakistan's predominant strain, especially in Karachi, where the incidence rate has approached 40% and no Pakistani city has been protected by the latest Omicron (2). In July 2022, healthcare authorities cautioned that the rise in new ailments may cause the pandemic's sixth wave (3, 4).

The first-line defensive strategy against COVID-19 is not feasible over the long run due to the global economic instability spurred on by travel restrictions and prolonged lockdowns, particularly in low-income nations like Pakistan where the invasion of new variations is foreseeable (5, 6). The most effective method to combat this catastrophe and prevent emerging mutations is immunization since it minimizes the possibility that the illness would be severe. Vaccination is fundamental to significantly reducing the impacts of COVID-19 infection and enabling children to resume regular lifestyles (7, 8). An effective immunization process requires both sufficient vaccine manufacturing and significant levels of acceptance. Exhilaratingly, over 100 potential vaccine options have been established since the virus's genomic structure was disclosed in January 2020 (9, 10). In the case of vaccinations that are 80% efficacious, statistical models suggest that 60–72% of the population must be immunized to achieve protective immunity (11). An increased vaccine acceptance may be essential in light of the advent of some novel variations with significant disease transmission, like the recently detected Omicron variant (12). Individuals over the age of 16 who get the BioNTech, Pfizer vaccine in a two-dose course are 95% protected against the emerging coronavirus variants (13). Nonetheless, Pakistan's immunization efforts are hindered by vaccine hesitancy, similarly to other underdeveloped nations. Between May 2021 and August 2022, almost 60% of Pakistan's population received two doses of immunization, while about 20% received booster shots (14).

Immunization reluctance is impeding this nation's vaccination effort. In Pakistan, vaccine reluctance has long been a significant phenomenon and a continuing obstacle to polio immunization efforts (15). Whilst investigations on the COVID-19 vaccine are underway, one significant barrier to vaccination may be vaccine hesitancy, which is described by the WHO as the delay in accepting or refusing immunization despite the accessibility of vaccination facilities (16). From the beginning of this catastrophe, there has been an increase in the misinformation concerning COVID-19 and its immunization that has been fostered by various conspiracies. Low socioeconomic position, inadequate vaccination administration methods, non-compliance, and lack of access to the vaccine are a few more variables that significantly contribute to this hesitation. These and other considerations make the general populace reluctant to receive vaccinations. The average public is reluctant to receive vaccinations due to all of these reasons (17). The presently existing vaccination no longer protects against the new N501Y strain due to spiking in protein mutation which is more infectious than the preceding variants as a result of the mutation. According to reports, the N501Y mutation is detected in about 25% of the recent incidences in Pakistan (18, 19). Professionals and scientists recommend a yearly booster dose to adequately prevent the emerging virus's potential to rapidly mutate to provide immunity against the recent N501Y variant. An updated formulation of the vaccine is now being designed and will probably be offered as a booster dose (20). However, this might make it considerably challenging for Pakistani healthcare authorities to persuade individuals to receive this additional yearly booster dosage. This mutation, though, might not be the last one the world experiences. The corona virus is anticipated to change regularly in the future, just like all other viruses, making yearly booster doses the primary strategy to maintain a sufficient level of immunity against this catastrophic infection (21, 22). In addition to having a devastating effect on the economy and healthcare infrastructures, this plague culminated in the suspension of polio vaccination programs, which contributed to an increase in instances of the disease (23).

According to epidemiological statistics, the most prevalent method of transmission tends to be aerosols from face-to-face exposure while sneezing or coughing (24). Healthcare workers are susceptible to this extremely contagious virus since they frequently come into touch with COVID-19 patients. Appropriate and reliable preventative vaccinations were thereby a potentially helpful weapon that potentially is adopted to minimize transmission rates and consequent illnesses in response to the outbreak (25, 26). There have recently been instances of corona infection, hospitalization, and fatalities in some patients who had received both vaccine shots (27). This introduces additional hurdles for frequent outbreak prevention and management. The COVID-19 outbreak is still threatening and must be addressed carefully. Furthermore, coronavirus has become vulnerable to mutation, and vaccination efficacy has declined over time, which is likely to have caused the pandemic's resurgence (27, 28). Thus, timely immunization with the third booster dose to additionally enhance the body's level of neutralizing antibodies can augment and strengthen the vaccine's waning protective effectiveness while also protecting a potential future coronavirus variation.

Healthcare workers in this instance are more likely to contract the ongoing virus than the general public (29–31). To build herd immunity among all the populations that might lead to COVID-19 transmission, healthcare workers must be immunized. This would prevent the propagation of the virus and have positive knock-on effects on the larger population. The majority of research on the COVID-19 booster shots to date has focused on examining people's willingness to receive them, with limited emphasis on the underpinning mechanism. Among healthcare workers, understanding the rationale of the willingness to acquire booster shots is imperative to enhance the immunization for booster shots in the general public, which could help combat the epidemic.

It is fundamental to increase vaccination trust among Pakistani people, which are primarily comprised of rural regions and repressed females. As a result, engagement and counseling are required to increase their confidence while addressing socioeconomic inequities in the population. The healthcare authority must ramp up its endeavors to confront this challenging catastrophe. To fight back, evidence-based communication, electronic media platforms, and legislative actions must be implemented. Widespread misconceptions about vaccination have to be countered by thorough informational analysis by technology or communications experts and the dissemination of opposing views from medical experts. Fears of the general population can be addressed with targeted and focused solutions; otherwise, the effects could last for centuries. The factors causing vaccination hesitancy must be remedied promptly to prevent Pakistan from combating the COVID-19 pandemic in vain. Vaccines and upcoming yearly booster injections are Pakistan's sole defense against a recurrence of COVID-19 infections. Thus, immunization hesitancy poses a significant barrier to effectively controlling the epidemic, prolonging it indefinitely and bringing about immeasurable pain and fatalities.

Earlier investigations revealed that discomfort at the vaccination spot, muscles and bone pains, overall unpleasant sensations, and fever were the most often reported adverse responses following immunization (32, 33). Investigating whether these unfavorable post-vaccination effects impact people's decision to accept booster doses is a topic of significant interest. People sometimes have to make unpleasant decisions regarding their health, and they might come to regret their actions in the future. One of the potential variables most commonly indicated to be connected to regretting decisions, according to the study, is a negative bodily health outcome (32, 34). In the present research, we thus sought to investigate the relationships between post-vaccination adverse effects, decision regret, and readiness to receive the COVID-19 booster dosage.

## Materials and methods

### Data source and study design

Between March 2 and April 31, 2022, we conducted a cross-sectional online survey. A web-based questionnaire was generated using Google-based forms and shared with the study participants *via* social platforms and e-mails. In accordance with the government's social distancing imperatives, face-to-face meetings were eliminated. The targeted populace included all healthcare employees in a public hospital in Rawalpindi, Pakistan, and the samples comprised clinicians, nurses, and technicians, as well as administrative staff. After performing a preliminary assessment of the collected data, repeated samples and respondents < 18 years were eliminated from the final sample. With a response rate of 41%, 1,343 responses were aggregated overall. There were three sections in the survey questionnaire which are described in Table 1.

**Table 1:** The main content of the study questionnaire adapted for the proposed study.

**Questionnaire contents**	
• Basic demographic characteristics	Age, gender, educational level, professional status, occupation, and any comorbidity
• Vaccination history	COVID-19 vaccination status Post-vaccination adverse effects (Yes or No)
• Decision regret (35)	a)The decisions were right. b)I regret the choices that were made. c)I would go for the same choice if I had to do it over again. d)The choices did me a lot of harm. e)The decisions were wise ones.
• Willingness to receive the booster dose against COVID-19	Yes or No

For items, a, b, c, d, and e, each was evaluated on a five-point bipolar intensity scale. Participants evaluated the statements by circling a number from 1 (strongly agree) to 5 (strongly disagree). Item b and d were phrased in the negative to avoid acquiescence bias. After reversing the score of these two items, the overall sum of the score was produced by taking the sum of the five items.

### Ethical consideration

This survey study was approved by the Ethics Committee of Benazir Bhutto Hospital, Punjab Province, Pakistan (*Ref: BBH-2021/004583*). All procedures were conducted in accordance with the guidelines of our institutional Ethics Committee and in compliance with the principles of the Declaration of Helsinki. Prior to completing the questionnaire forms, all participants provided their electronic informed consent. By maintaining anonymity throughout the investigation and requesting genuine responses and choices from the participants, the integrity of the data was well-preserved.

### Mediation analysis

Several researchers have adopted mediation frameworks to investigate the prospective influence of an independent parameter on a response factor and to determine if a parameter may have a mediation connection among different parameters (36, 37). Since mediation analysis is vital in comprehending the process by which an alteration in one factor may lead to an alteration in another, this research may have significant policy implications. In the present study, exposure (*X*) represented post-vaccination adverse effects (either in “Yes” or “No”); a putative mediator (M) represented regret over a decision, and the outcome (Y) represented the likelihood to receive a COVID-19 booster shot. We emphasized the scenario of a continuous mediator (M) and a dichotomous event (Y), and we employed the three regression packages listed below for mediation analysis:


(1) $$\begin{array}{l} logit\left(P\left(Y=1\right)\right)=C_{1}+\text{λ}X+\mu^{T}Z+\epsilon_{1} \end{array}$$

(2) $$\begin{array}{l} M=C_{2}+\text{δ}X+\phi^{T}Z+\epsilon_{2} \end{array}$$

(3) $$\begin{array}{l} logit\left(P\left(Y=1\right)\right)=C_{3}+\lambda^{*}X+\eta M+\phi^{T}Z+\epsilon_{3} \end{array}$$

Here, Equation (1) shows how an explanatory and a dependent variable (*i*.*e*., *X*, and Y) are related; the relationship between an explanatory parameter and a mediator (*i*.*e*., *X*, and M) is described by Equation (2). The interrelationship between the explanatory parameter, the mediator, and the dependent factor is described in Equation (3). Whereas, ‘Z' represents the covariates including age and gender; λ represents the overall influence of the exposure on the outcome; δ represents exposure influence on the mediator; λ^*^ represents the exposure's direct influence on the outcome; η represents the mediator effect on the outcome. Also, the intercept terms are represented by $C_{1}$, $C_{2}$, and $C_{3}$ were the intercept terms while the residual terms are represented by ϵ_1_, ϵ_2_, *and ϵ*_3_.

In the existing literature, the most widespread strategy for testing mediation interactions has been regression-based modeling (38–41). Finding out whether *X* and Y had a meaningful relationship was the first step. The significance of the relationship between *X* and M was examined in the subsequent stage. Regressing Y on *X* and M was the ultimate step. Lastly, we employed the paired analysis technique to examine the mediation effect (42). This approach took into account the path-specific *P*-values and yielded the following estimation:


(4) $$\begin{array}{l} \mathbb{P}=\max\left({\mathbb{P}}_{\delta }{,\;\mathbb{P}}_{\eta }\right) \end{array}$$

In the case of *p* < 0.05, we may therefore assume that the variable “M” served as the intermediary between the explanatory (*X*) and the outcome (Y).

## Results

Table 2 describes the participants' baseline demographics' such as age (years), educational level, occupation, gender, professional status, comorbid conditions as well as vaccination status and post-vaccination adverse responses. We retrieved 1,369 accurate responses, and 1,343 (98.10%) of those who responded had fulfilled their two shots of COVID-19 immunizations with a proportion of 9.23% experiencing adverse effects after receiving each dose. Approximately 40% of the healthcare participants fall in the age group 30–39 who completed their two shots of the COVID-19 vaccine. With a significant difference of 64.1%, females outweighed males among research participants. Among all, 61.88% of study participants were nurses, with a comparable proportion (65.60%) holding primarily undergraduate degrees. Furthermore, a significant number of participants (86.53%) did not have any underlying diseases.

**Table 2:** Baseline demographics of study participants (*n* = 1,343).

	**Frequency (*n*)**	**Percentage (%)**
**Age (years)**		
< 30	466	34.70
30–39	548	40.80
40–49	240	17.87
≥ 50	89	6.62
**Gender**		
Male	241	17.95
Female	1,102	82.06
**Educational level**		
Secondary or below	139	10.35
College	175	13.03
Undergraduate	881	65.60
Graduate	148	11.02
**Occupation**		
Clinicians	175	13.03
Nurses	831	61.88
Technicians	236	17.57
Administrative	101	7.52
**Professional status**		
Internees	36	2.68
Medical officer	1,029	76.61
Associate Professor	105	7.82
Professor	61	4.54
Others	112	8.34
**Any comorbidity**		
Yes	181	13.48
No	1,162	86.53
**Post-vaccination adverse effects**		
Yes	124	9.23
No	1,219	90.77

Comorbidity includes Diabetes mellitus; Hypertension; Chronic respiratory disease; cardiovascular disease; chronic kidney disease; Chronic liver disease and Cancer.

Table 3 displays the findings on the prevalence of willingness to receive the COVID-19 booster shot of healthcare workers. An aggregate of 1,236 (92.03%) research participants expressed their willingness to acquire the booster shot with a significant distinction (χ^2^ = 31.06, *p* < 0.000) between those who experienced post-vaccination adverse effects and those who did not. Only 76.55% of individuals who experienced negative side effects after the immunization was willing to get the booster dose, compared to 97.37% of those who did not. Furthermore, the findings of the univariate analysis revealed no significant differences in expressing willingness against COVID-19 immunization across age, gender, educational level, occupation, professional status, and underlying comorbid conditions. In certain categories, we noticed a higher acceptance rate of the vaccine, though. For instance, 96% of participants over 50 years indicated their willingness toward the booster shot. Research participants with a college degree, doctors, professors, and those without underlying conditions were also more inclined to administer the booster dosage.

**Table 3:** Univariate analysis of baseline characteristics of participants and willingness to take the booster shot against COVID-19 infection.

	**Frequency**	**Percentage**	**χ^2^**	***P*-value**
**Total**	1,343	92.03		
**Age (years)**			3.78	0.548
< 30	466	86.19		
30–39	548	84.37		
40–49	240	90.14		
≥ 50	89	96.00		
**Gender**			0.163	0.602
Male	241	83.50		
Female	1,102	89.69		
**Educational level**			5.20	0.507
Secondary or below	139	88.16		
College	175	90.05		
Undergraduate	881	86.03		
Graduate	148	85.39		
**Occupation**			8.10	0.063
Clinicians	175	78.90		
Nurses	831	84.39		
Technicians	236	91.48		
Administrative	101	88.75		
**Professional status**			2.69	0.552
Internees	36	81.94		
Medical officer	1,029	83.29		
Associate Professor	105	87.04		
Professor	61	97.07		
Others	112	91.11		
**Any comorbidity**			3.10	0.055
Yes	181	88.36		
No	1,162	90.41		
**Post-vaccination adverse effects**			31.06	**0.000**
Yes	124	76.55		
No	1,219	97.37		

The bold values indicate statistically significant at < 0.05.

Table 4 represents the descriptive statistics and correlation coefficients of the selected variables. The likelihood of accepting the booster shot was negatively correlated with post-vaccination adverse effects (*r* = −0.20;*p* < 0.001) and positively correlated with decision regret (*r* = 0.31;*p* < 0.001). While decision regret was found inversely associated with a participant's readiness to accept the booster shot (*r* = −0.27;*p* < 0.001). In conclusion, the correlation analysis findings demonstrated that the pairwise comparisons of the aforementioned three factors were substantial and highlighted that there was a relationship between post-vaccination adverse effects, intention to receive the booster shot, and decision regret.

**Table 4:** Descriptive statistics and correlation among study participants.

**Variables**	**Descriptive**	**1**	**2**	**3**
1. Post-vaccination adverse effect	124 (124%)	1.00		
2. Decision regret	9.1 (**±** 4.3)	0.31^***^	1.00	
3. Willingness to receive booster shot against COVID-19	1,236 (92.02%)	−0.20^***^	−0.27^^***^^	1.00

*** indicates p < 0.001. For the categorical variables, we computed frequency and percentages. For continuous variables, we expressed in ± S.D.

### Testing for the mediation model

Table 5 outlines the findings of the mediation analysis that consider the controlled variables (age, gender, educational attainment, occupation, and comorbid conditions) to determine the correlation between post-vaccination adverse effects, inclination to consider taking the booster shot, and decision regret. The first finding showed that individuals' willingness to receive the booster dosage was significantly impacted by post-vaccination adverse effects (*p* < 0.001). Participants who experienced post-vaccination adverse effects were less likely to obtain the booster shot than those who did not [*OR* = 0.41;*CI*(0.21 − 1.69)]. Therefore, the post-vaccination adverse effects was a key element influencing the decision to accept the booster shot. Secondarily, participants who encountered adverse responses had stronger decision regret levels than participants who did not experience post-vaccination adverse effects [β = 1.54;*CI*(1.01 − 2.61)]. The unfavorable experience following immunization had a considerable impact on decision regret (*p* < 0.001).

**Table 5:** Evaluating decision regret as a mediating factor.

**Characteristics**	**MODEL−1**	**MODEL−2**	**MODEL−3**
	***OR (95% CI)***	**β** ***(95% CI)***	***OR (95% CI)***
**Independent variable**			
*Post–vaccination adverse effects (No)*			
Yes	**0.41** (0.21–1.69)	**1.54** (1.01–2.61)	**0.52** (0.38–1.58)
**Mediator**			
*Decision regret*			**0.82** (0.76–0.91)
**Controlled variables**			
*Age (< 30)*			
30–39	2.24 (2.01–3.47)	−0.32 (−0.13–0.93)	2.00 (1.50–3.76)
40–49	2.91 (1.63–4.01)	−0.65 (−0.33–0.68)	2.64 (1.94–4.55)
≥ 50	3.35 (2.76–10.28)	−0.25 (−1.19–0.57)	3.09 (2.98–9.63)
**Gender (Male)**			
Female	0.69 (0.39–1.96)	0.23 (−0.76–2.08)	0.74 (0.45–2.39)
**Educational level (Secondary or below)**			
College	**3.72** (2.52–9.76)	−**1.59** (−4.52–0.11)	2.42 (1.04–7.66)
Undergraduate	1.89 (0.38–5.19)	−**2.08** (−3.77–1.42)	1.38 (0.74–5.07)
Graduate	2.14 (0.88–7.34)	**−3.31** (−3.31–0.98)	1.27 (0.23–6.59)
**Professional status (Clinicians)**			
Nurses	3.43 (2.94–5.09)	−0.68 (−1.79–0.53)	3.17 (1.79–4.49)
Technicians	**5.47** (3.37–11.28)	**−1.42** (−2.67–1.70)	**4.30** (3.18–9.61)
Administrative	4.77 (2.19–8.45)	0.32 (0.97–1.75)	5.00 (3.89–9.17)
**Any comorbidity (No)**			
Yes	**0.72** (0.52–2.79)	−0.11 (−0.89–1.46)	**0.67** (0.48–2.32)

Bold shows statistically significant at < 0.001, 0.01, and 0.05. Model 1 and model 3 show willingness of acquiring the booster shot against COVID−19 (1 denotes “Yes”); the model 2 outcome shows decision regret. OR; odds ratio, CI: confidence interval, β: standardized beta regression coefficient.

The bold values indicate statistically significant at < 0.001, 0.01, and 0.05.

After adjusting for post-vaccination adverse effects, it was also discovered that the influence of decision regret on willingness to receive the booster shot was significantly correlated [*OR* = 0.82;*CI*(0.76 − 0.91)], suggesting that respondents who regretted their prior considerations were less likely to receive a booster shot. Last but not least, the unfavorable experience after vaccination continued to have a substantial impact on willingness to get the booster shot [*OR* = 0.52;*CI*(0.38 − 1.58)]. Age, gender, educational level, occupation, and underlying comorbid were all taken into account while adjusting for all models. The technicians were more inclined to take the booster dosage than doctors were. Additionally, individuals with underlying disorders were less inclined to receive the booster shot than those without underlying diseases. The outcomes of the combined investigation showed that the association between post-vaccination adverse effects and willingness to receive the booster dosage was significantly mediated by decision regret (ℙ = max(ℙ_δ_, ℙ_η_) < 0.05). This demonstrates that regret about a decision may greatly moderate the influence of negative post-vaccination adverse effects on motivation to get the booster dosage.

## Discussion

The COVID-19 outbreak has had a tremendous detrimental effect on people's health all across the world and has resulted in significant illness and economic difficulties. Immunization is witnessed as a reliable and secure approach to preventing and controlling infectious diseases. Given the likelihood that the corona infection may prolong to affect humans, we may need to be equipped for continuous immunizations. The healthcare immunization program in Pakistan is systematic, and significant immunization rates among healthcare workers are projected, unfortunately, research on the probable causes underlying the willingness to accept the booster dosage in Pakistan is inadequate.

The purpose of the present investigation was to preliminary investigate the association between post-vaccination adverse effects and intention to accept the booster dosage, as well as the putative operations involved. We emphasized healthcare workers who've already done the two-step immunization protocol. We determined that individuals who experienced post-vaccination adverse responses had a negative connection with their acceptability of the booster shot. Likewise, participants who had negative responses to vaccinations were more inclined to regret their earlier vaccination decisions. Furthermore, those with increased levels of decision regret were less inclined to accept the booster shot. According to the findings, regret about earlier decisions might greatly mitigate the influence of post-vaccination adverse effects on the intention to undertake the booster shot. The present research may be one of the few investigations on the impact of post-vaccination adverse effects on intent to undergo a booster shot within the Pakistani context.

There is compelling evidence that the COVID-19 booster shot may boost the titer and defensive spectrum of neutralizing antibody levels. Considerable work has been done to explore the immunogenicity, safeness, and effectiveness of the COVID-19 booster shot (43–45). The study's findings revealed that 92.02% of the healthcare workers were willing to take the booster shot, which was less than the stated acceptance proportion of the main immunization in earlier studies conducted in Pakistan (46–48). Additionally, considerable attention has been focused on the consequences of various vaccination rate factors (48–50). Evidence from Chile revealed that respondents, including researchers and medical experts, who trusted COVID-19 vaccinations were substantially more prepared to receive the booster dosage (48). Likewise, doctors and nursing healthcare employees in Singapore showed a reduced median time before obtaining a COVID-19 booster shot than their organizational and allied health counterparts (50). According to earlier research, around one-fourth of those who were immunized, irrespective of the vaccine, experienced negative effects after receiving the shot (32). The decision to acquire the vaccination could be regretted even though all adverse effects fade away after a week. One of the risk variables most commonly indicated to be connected to regretful decision-making was worse physical health results (51). The adverse repercussions from the vaccination as well as decision regret made people less inclined to have the booster shot. Consequently, it is imperative to integrate knowledge regarding adverse responses after vaccination in ongoing vaccine awareness campaigns and policy measures that support COVID-19 booster shots. Doing so may enhance the willingness to get immunized (52). The general population, which intently observes how healthcare personnel behave on this situation, may embrace vaccination more readily if more healthcare workers are motivated to get immunized against corona infection People are more inclined and willing to get immunized if healthcare personnel encourage it, hence healthcare workers' perspectives are especially crucial for vaccine adoption in the general population. The vaccination advocacy across academics and healthcare facilities is an additional added value of strengthening vaccination programs (53, 54). Public health authorities should emphasize offering more plausible information on the COVID-19 outbreak, particularly regarding possible consequences after being immunized, as well as convincing and perhaps forcing healthcare workers to get immunized against coronavirus infection.

## Limitations

A few limitations of our investigation must be considered when interpreting the results of our investigation. To begin, as we only evaluated one territorial hospital, the chosen sample may not be profoundly indicative of Pakistani healthcare employees. Secondarily, considering that they were in relatively good health to function in a healthcare facility, survey respondents were probably healthier than the general population, which could have led to selection bias. Thirdly, the great majority of research participants were healthy young adults without comorbid conditions. A more reliable assessment would be achieved from more research that balance these demographic factors. Also, we emphasized the healthcare workers who had finished their double immunization. There could, however, be variations between healthcare workers and the local population. The generality and external reliability of the information and outcomes should thus be extensively investigated to better understand the influence of decision regret in the association between unfavorable responses after Immunization and intention to undertake the booster shot. Additionally, the web-based data collection approach had limitations that could have caused participants to over- or under-report their willingness to accept the booster dosage. Lastly, our projections were made at a single time point and were not adjusted for long-term exposure to multiple influences. Additional longitudinal studies with considerably larger sample populations are anticipated not only to generalize conclusions to other regions of Pakistan, in addition, to thoroughly comprehend the causative interconnections.

## Conclusion

In summation, our outcomes suggest that post-vaccination adverse repercussions for healthcare workers may impair their willingness to undertake the COVID-19 booster shot. Notably, healthcare workers who had unfavorable experiences with vaccination were increasingly inclined to regret their earlier vaccination decisions, which restricted their inclination to acquire a booster dose even further. These outcomes demonstrate valuable information for enhancing the immunization ratio of booster shots in the future, albeit participant bias should be taken into account. The majority of post-vaccination adverse consequences fade away within a week, so immunization programs shouldn't be overly concerned about them. To enhance vaccine acceptance intent and perhaps boost intention to acquire booster shots against COVID-19 disease, post-vaccination negative impacts should be more thoroughly incorporated into vaccine awareness programs and policy initiatives that advocate additional doses.

## Data availability statement

The raw data supporting the conclusions of this article will be made available by the authors, without undue reservation.

## Ethics statement

The studies involving human participants were reviewed and approved by the Ethics Committee of Benazir Bhutto Hospital, Punjab Province, Pakistan (Ref: BBH-2021/004583). The patients/participants provided their written informed consent to participate in this study.

## Author contributions

All authors listed have made a substantial, direct, and intellectual contribution to the work and approved it for publication.

## Funding

This work was supported by the Union Program of Science and Health of Hunan Province, China (2019JJ80011). The funder had no role in study design, data collection, analysis, decision to publish, or preparation of the manuscript.

## Conflict of interest

The authors declare that the research was conducted in the absence of any commercial or financial relationships that could be construed as a potential conflict of interest.

## Publisher's note

All claims expressed in this article are solely those of the authors and do not necessarily represent those of their affiliated organizations, or those of the publisher, the editors and the reviewers. Any product that may be evaluated in this article, or claim that may be made by its manufacturer, is not guaranteed or endorsed by the publisher.
